# Differential sialotranscriptomes of unfed and fed *Rhipicephalus haemaphysaloides*, with particular regard to differentially expressed genes of cysteine proteases

**DOI:** 10.1186/s13071-015-1213-7

**Published:** 2015-11-17

**Authors:** Xinmao Yu, Haiyan Gong, Yongzhi Zhou, Houshuang Zhang, Jie Cao, Jinlin Zhou

**Affiliations:** Key Laboratory of Animal Parasitology of Ministry of Agriculture, Shanghai Veterinary Research Institute, Chinese Academy of Agricultural Sciences, Shanghai, 200241 China; Jiangsu Co-innovation Center for Prevention and Control of Important Animal Infectious Diseases and Zoonoses, Yangzhou, 225009 China

**Keywords:** Sialotranscriptome, *Rhipicephalus haemaphysaloides*, Cysteine proteases, Dynamic change

## Abstract

**Background:**

*Rhipicephalus haemaphysaloides*, a hard tick, is a common ectoparasite and can be found in many countries. It is recognized as the primary vector of bovine babesiosis in the south of China. During blood feeding, the tick’s salivary glands secret numerous essential multifunctional proteins. In this study, a *R. haemaphysaloides* salivary gland transcriptome was described following the production and analysis of the transcripts from the two cDNA libraries of unfed and fed female ticks. The study focused on the differentially expressed genes and cysteine proteases, which play essential roles in the tick life cycle, that were detected most commonly in the up-regulation libraries.

**Methods:**

The sialotranscriptome was assembled and analyzed though bioinformatic tools and the cysteine protease which is differentially expressed form sialotranscriptome were confirmed by Real-time PCR in salivary glands and different developments of ticks.

**Results:**

On the basis of sequence similarities with other species in various databases, we analyzed the unfed and fed sialotranscriptome of *R. haemaphysaloides* to identify the differentially expressed proteins secreted from the salivary glands during blood feeding and to investigate their biological functions. There were 25,113 transcripts (35 % of the total assembled transcripts) that showed significant similarity to known proteins with high BLAST from other species annotated. In total, 88 % and 89 % of the sequencing reads could be mapped back to assembled sequences in the unfed and fed library, respectively. Comparison of the abundance of transcripts from similar contigs of the two salivary gland cDNA libraries allowed the identification of differentially expressed genes. In total, there were 1179 up-regulated genes and 574 down-regulated genes found by comparing the two libraries. Twenty-five predicted cysteine proteases were screened from the transcript databases, whereas only six protein molecules were confirmed by gene cloning and molecular expression in *E.coli* which all belonged to the cysteine protease family. Bioinformatic evolutionary analysis showed the relationship of cysteine proteases in ticks with those of other species, suggesting the origin and conservation of these genes. Analysis of sequences from different tick species indicated the further relationships among the proteases, suggesting the closely related function of these genes. Thus, we confirmed their changes in unfed, fed and engorged ticks and salivary glands. The dynamic changes revealed their important roles in the tick life cycle.

**Conclusions:**

Our survey provided an insight into the *R. haemaphysaloides* sialotranscriptome. The dynamic changes of cysteine proteases in ticks will assist further study of these proteases, which may contribute to the development of anti-tick vaccines or drugs, as well as improving understanding of the roles of cysteine proteases in the tick life cycle.

**Electronic supplementary material:**

The online version of this article (doi:10.1186/s13071-015-1213-7) contains supplementary material, which is available to authorized users.

## Background

As a kind of blood-feeding arthropod, ticks can transmit viruses, bacteria and protozoa in their meals [1]. Up to now, there are over 800 species described worldwide which are classified in Ixodidae (hard ticks) and Argasidae (soft ticks) primarily [2]. *Rhipicephalus haemaphysaloides* is a three-host tick belonging to the Ixodidae and is widely distributed in China, India, and other South Asian countries [3]. This tick is a major vector of bovine babesiosis in China [4] and can also transmit the Kyasanur Forest disease virus [5].

Within the blood-feeding, ticks possess salivary glands that secrete bioactive substances, which can exhibit a range of pharmacological properties to thwart the host defense mechanisms in response to attachment [6, 7]. The components of the saliva are of major importance for the tick’s survival, helping it feed and evade host defenses, hemostatic factors and the inflammatory response [8]. Proteases are one of the most important components of tick saliva and essential for the life cycle of the ectoparasite.

Cysteine proteases are ancient conserved proteases that are involved in different physiological processes [9]. Most of these proteases belong to the papain-like superfamily and are associated with the development of hematophagous arthropod ectoparasites [9]. Ticks express cysteine peptidases with important roles in physiological events that are crucial to the ectoparasitic lifestyle, including the digestion of host blood, embryogenesis and innate immunity [9].

In this study, we analyzed the sialotranscriptome of the salivary glands of unfed (unattached) and fed (3 or 4 days after attached) adult ticks (Additional file 1). There were 1179 up-regulated genes and 574 down-regulated genes detected from the differential expression databases. For functional annotation of the unique transcripts, we used BLASTx, comparing them against different databases and, finally, four up-regulated and two associated cysteine proteases, namely cathepsin B (CATB, KT194088), cathepsin L (CATL, KT194089), caspase–1 (CASP1, KT194090), caspase–8 (CASP8, KT194091), autophagy protease 4B (ATG4B, KT194092) and autophagy protease 4D (ATG4D, KT194093), were cloned successfully from the cDNA library of the salivary glands of *R. haemaphysaloides*. The confirmation of these genes will contribute to further research in vitro. The dynamic analysis of these target genes was shown in different developmental stages of *R. haemaphysaloides* by Q-PCR, and suggests their important roles during blood feeding. To our knowledge, this is the first analysis of the transcriptome of the salivary glands of female *R. haemaphysaloides* ticks. The characterization of the components of tick saliva, especially the proteases (cysteine proteases), is likely to be of value in the design of novel methods or drugs for the control of ticks and tick-borne diseases, as well as when searching for proteins that may have potential use in research on medical and veterinary diseases.

## Methods

### Collection of ticks and salivary glands

The *R. haemaphysaloides* colonies were maintained in the laboratory as described previously [10]. For tissue collection, the salivary glands were dissected under a light microscope [10]. The sample materials were stored at −80 °C until use.

### cDNA library construction and sequencing

Total RNA was extracted from the unfed and fed salivary glands of female *R. haemaphysaloides* using TRIzol Reagent (Invitrogen, The Netherlands) according to the manufacturer’s protocol. The cDNA from two RNA-seq sequencing libraries was sheared to an average fragment size of about 300 values and was purified with Ampure beads. RNA-seq libraries were constructed according to the Illumina manufacturer’s instructions for 100-bp paired-ends, and sequenced. Raw reads were filtered to produce clean reads prior to assembly. Initially, the RNAs were extracted and constructed for two Illumina/Solexa cDNA libraries.

### Data analysis

#### *De novo* assembly of transcriptome data

Low-quality regions in raw reads and adaptors were trimmed with ea-utils [11] prior to analyses. Read quality was then assessed, revealing that the mean quality scores of sequence reads was around 37 values, which suggested high sequencing quality. Transcriptome assembly was performed with Trinity assembles software [12] to obtain high-quality contigs. The Trinity assembly program first combines reads with a certain length of overlap to form longer fragments without N; these are called contigs. The sequencing reads are then mapped back to contigs; with paired-end reads it is able to detect contigs from the same transcript as well as measuring the distances between these contigs. Subsequently, Trinity connects the contigs using N to represent unknown sequences between each pair of contigs, following which Scaffolds are made.

#### Bioinformatic tools used and differential expression analysis

The BLASTn [13], CAP3 assembler [14] and ClustalW software [15] were used to compare, assemble, and align high quality expressed sequence tags, ESTs, respectively. For functional annotation of the transcripts we used BLASTx [13] to compare the nucleotide sequences with the non-redundant (NR) protein database of the National Center of Biological Information (NCBI) and to the Gene Ontology (GO) database [16]. The gene expression profiles were compared by mapping RNA-seq reads using Bowtie 2 2.1.0 [17]. Assembled sequences with high BLAST similarity to known protein sequences (E-value cut-off of 1E-6) from other species were annotated and the GO functional annotations were extracted using the Swiss-Pro (http://www.uniprot.org/) BLAST result by comparing with EMBL Uniprot eggNOG/GO Pathways databases. Analysis of GO terms was subsequently performed using a custom script. The GO terms belonging to cellular components, biological processes and molecular functions were listed. Bi-directional best hit (BBH) was used to search against the KEGG database [18] to obtain the KO (Reference pathway) number of the KEGG Annotation [18]. The KO (Reference pathway) number of the transcriptome was also obtained, according to KEGG Annotation.

To detect the changes in global gene expression in the different tissues, we applied the MA-plot-based method with a random sampling model [19] to identify the differentially expressed genes by comparing the unfed library with the fed library. Genes with fold change >3 and *P* value <0.001 were regarded as differentially expressed genes.

### Analysis of relative expression by quantitative real-time PCR

Total RNAs were purified from different developmental stages of ticks (eggs, larvae, nymphs and adult females) and from unfed (unattached), fed (3 or 4 days after attached) and engorged (blood feeding completely) female tick salivary glands. The cDNAs were synthesized from 200 ng RNAs using random 6-mer primers with the PrimeScript RT reagent kit (Perfect Real Time) (Takara, Shiga, Japan) in the following program: 37 °C for 15 min, 85 °C for 7 s, and finally 4 °C. Quantitative real-time PCR was performed using SYBR Premix Ex Taq (Takara, Japan) with a StepOnePlus Real-Time PCR System (Applied Biosystems, USA), with cycling parameters of 95 °C for 30 s, followed by 40 cycles of 95 °C for 5 s and 60 °C for 30 s. The primers are listed in Table 1. Gene-specific standards were the respective plasmids. All samples were analyzed three times.

**Table 1:** The Primers used in real-time PCR

Target gene	Forward primer (5′-3′)	Reverse primer(5'-3')	GeneBank number
CAT B	GCACCACCATTGGCGAGATTC	CTCGTAGTTGCCGCCCGTG	KT194088
CAT L	CTGAGGGCTTTGAGGATTTGC	GACCACCCTCGCAGCCG	KT194089
ATG4B	GGCACCTTGGGAAAGACTGGC	GCGTCTGTTGTCTCCACTCTGCAT	KT194090
ATG4D	AAGCAGGCGGGTGACTGGTAT	AATGTATGTGGTGTTGAGCTGTTCC	KT194091
CASP1	TCCACGGTGCCAGGCTTCTAC	CCAGTCGGGTCAGAGTGGAGGAG	KT194092
CASP8	GCAGGCACGCTCTACCAGTC	GCTCCTCTCATACAGCAGCACTA	KT194093

The data were normalized to the elongation factor-1 gene (EF-1) (accession number AB836665) [20]. Relative gene expression data were analyzed using the 2^—△Ct^ method [21, 22], and △Ct values were calculated by subtracting the average EF-1 Ct values from those for the average target gene.

Mean ± SE values for each group (*n* = 4) were calculated, and two-tailed t tests were used to compare differences between groups with Graphpad PRISM 5 software (GraphPad Software Inc., La Jolla, California).

## Results

### Comprehensive data

In total, more than 83 million paired-end reads were obtained from Illumina/Solexa sequencing (Table 2). The transcriptome assemble yielded 71,539 transcripts belonging to 29,932 genes. There were 42,789 transcripts (60 % of the total assembled transcripts) which had a length greater than 1 kb and 25,113 transcripts (35 % of the total assembled transcripts) showed significant similarity to known proteins with high BLAST (E-value cut-off of 1E-6) from the other species annotated (Table 3).

**Table 2:** Statistics sequencing amount for two fastq files

Samples	Clean reasd	Total nucleotides	Sequence length	GC percentage
Unfed	46,707,727*2	9,341,545,400	100 nt*2	51 %
Fed	36,571,011*2	7,314,202,200	100 nt*2	50 %

Total Reads and Total Nucleotides are actually clean reads and clean nucleotides; Total Nucleotides = Total pair-end Reads1 x pair-end size + Total pair-end Reads2 x pair-end Read2 size; GC percentage is proportion of guanidine and cytosine nucleotides among total nucleotides

**Table 3:** The summary of transcripts of salivary glands of *R.haemaphysaloides*

Total number of reference transcripts	71,539
The number of transcripts with BLASTX-hit	25,113
The number of transcripts with KEGG annotation	4,723
The number of transcripts detected with RPKM	10,760(unfed)/10,928(fed)
The number of up-regulated genes	1179
The number of down-regulated genes	574
GO from up-regulated expressed genes	135
GO cellular component /molecular function /biological process (up-regulated expressed genes)	29/33/73
GO from down-regulated expressed genes	15
GO cellular component /molecular function /biological process (down-regulated expressed genes)	5/9/1

*RPKM* Reads Per Kilobase of exon model per Million mapped reads, *KEGG* Kyoto Encyclopedia of Genes and Genomes, *GO* gene ontology

**Fig. 1:**
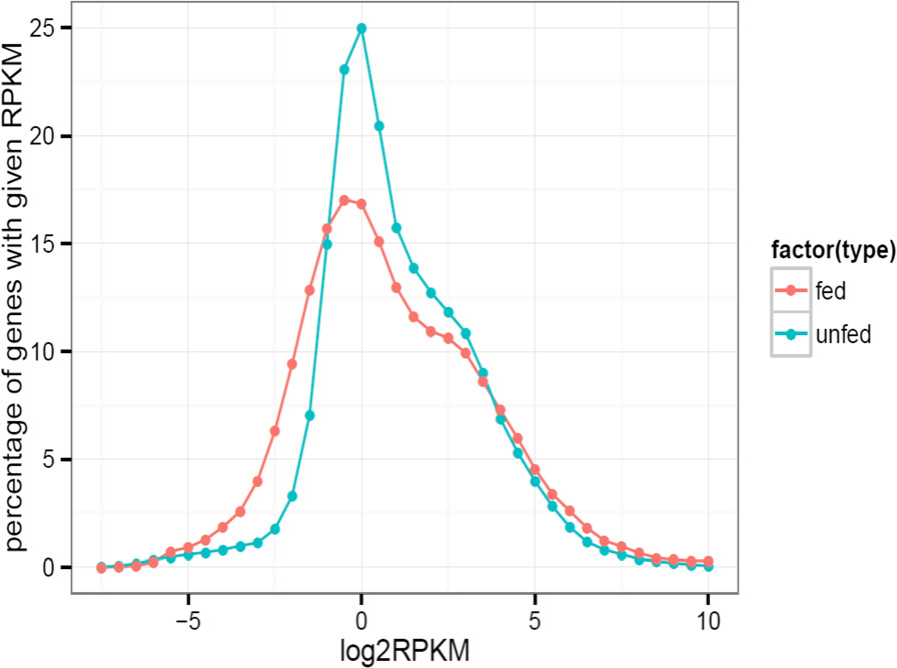
Distribution of RPKM values

In total, 88 % and 89 % of the sequencing reads could be mapped back to assembled sequences in the unfed and fed library, respectively. The raw count for each assembled sequence was calculated on the basis of the alignment files, and the gene expression levels were measured and normalized as reads per kilobase of the exon model per million mapped reads (RPKM) [23], which indicates that the total transcripted region of a gene has been detected. The distribution of expression values for the unfed and fed libraries was examined (Fig. 1).

**Fig. 2:**
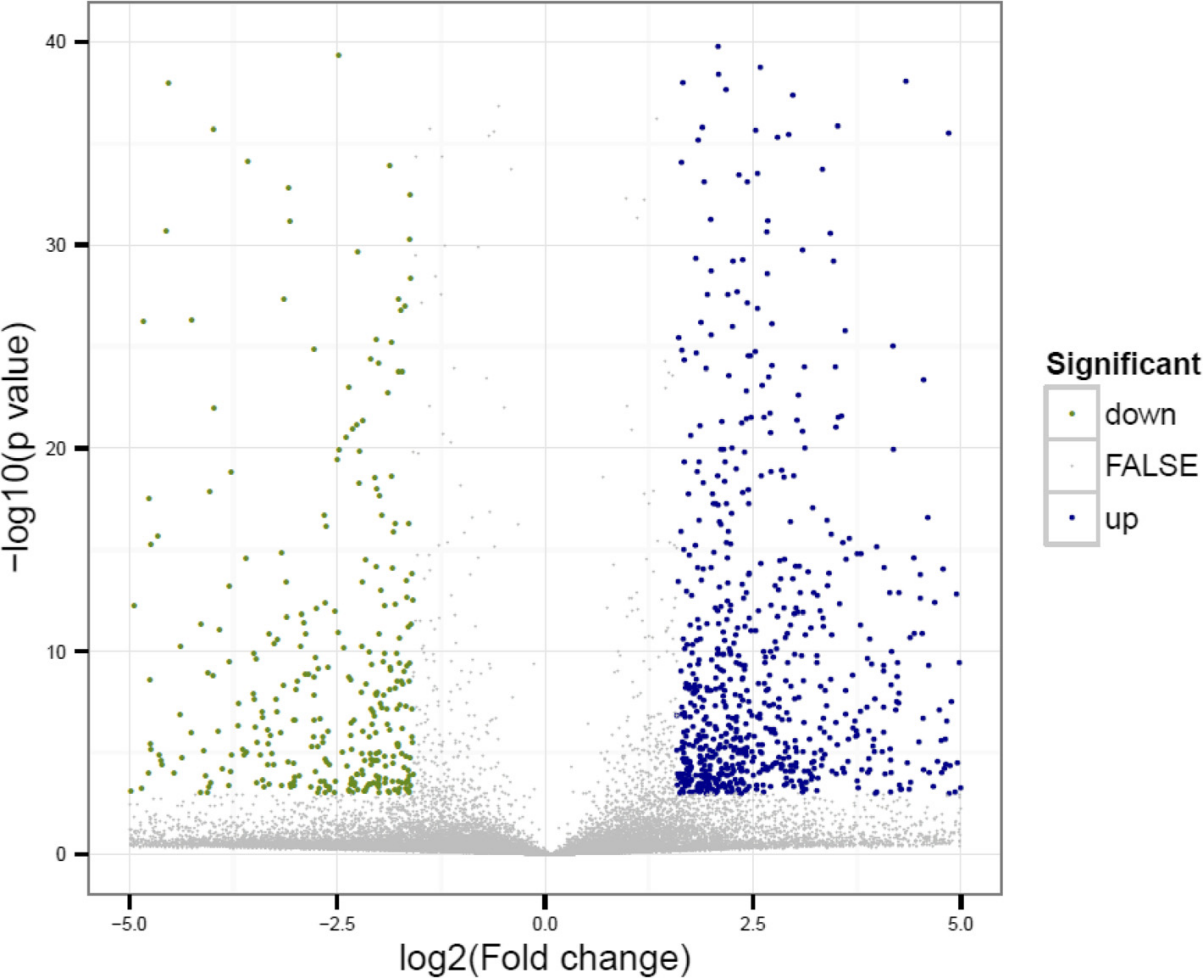
Volcano plots of differential expressed genes. A scatter plot showing differentially expressed genes. The X axis and Y axis show the fold changes on a log2 scale and the *p*-value on –log10 scale, respectively. The up-regulated genes and down-regulated genes are shown in green and blue, respectively

The average RPKM value in the fed and unfed mutant libraries was 20 and 21, respectively. In total 10,760 and 10,928 genes were detected that had RPKM >3 in the unfed and fed library, respectively (Table 3). Low-abundance transcripts were also detected by the RNA-seq, which suggested that the RNA-seq in this study provided high resolution for detecting the level of gene expression. In total, 1179 up-regulated genes and 574 down-regulated genes were found by comparing the two libraries (Table 3 and Fig. 2).

To investigate the functions of these differentially expressed genes, all the differentially expressed genes were mapped to the GO database and compared with the whole transcriptome background. BiNGO [24] was used to identify enrichment GO terms by using custom annotation files from the transcriptome on the basis of the hypergeometric test (*P*-value <0.01) (Additional file 1). All the enrichment GO terms from the up-regulated expressed genes could be categorized into 135 GO terms (29 cellular component terms, 33 molecular function terms, and 73 biological process terms). The enrichment GO from down-regulated expressed genes could be categorized into 15 GO terms (5 cellular component terms, 9 molecular function terms, and 1 biological process term) (Table 3).

Among all the GO terms there were 7 annotations which contained 39 contigs related to protease (Table 4). The transcript databases showed 25 predicted molecules that may have the molecular function of cysteine peptidase activity, on the basis of molecular function GO terms (GO:0008234).

**Table 4:** The summary of GO terms about proteases

GO term	Forecast function
GO:0002020	protease binding
(The number of related gene)	(3)
GO:0004843	ubiquitin-specific protease activity
(The number of related gene)	(29)
GO:0016505	apoptotic protease activator activity
(The number of related gene)	(1)
GO:0016929	SUMO-specific protease activity
(The number of related gene)	(3)
GO:0019783	small conjugating protein-specific protease activity
(The number of related gene)	(1)
GO:0019784	NEDD8-specific protease activity
(The number of related gene)	(1)
GO:0035800	ubiquitin-specific protease activator activity
(The number of related gene)	(1)

### Bioinformatic evolutionary analysis

**Fig. 3:**
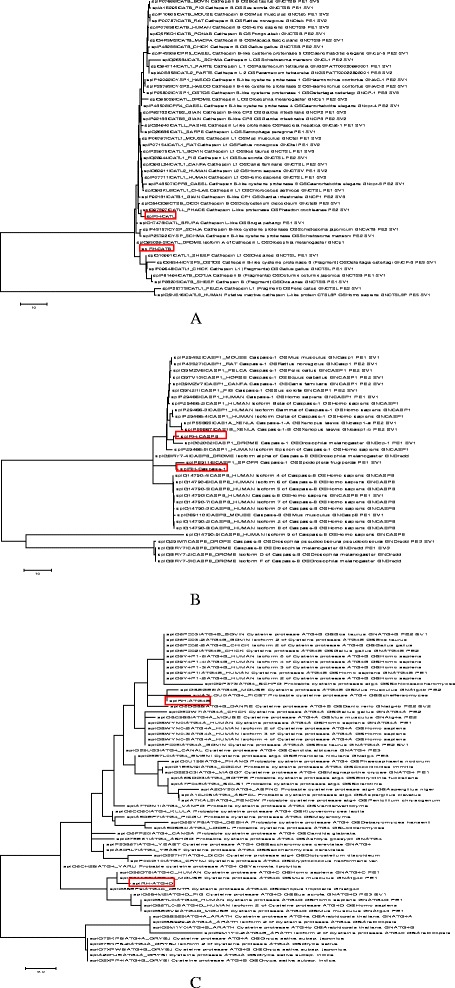
Relationship of *Rhipicephalus haemaphysaloides* cysteine protease (marked by red box) to other species proteins. **a**, the evolution of cathepsins (mainly cathepsin B, cathepsin L and their associated proteins); **b**, the evolution of caspases; **c**, the evolution of autophagy proteins. The circular phylogram is based on the alignment of sequences derived from this study using MEGA by maximum likelihood and similar sequences obtained from the Swiss-Prot database from UniProt

**Fig. 4:**
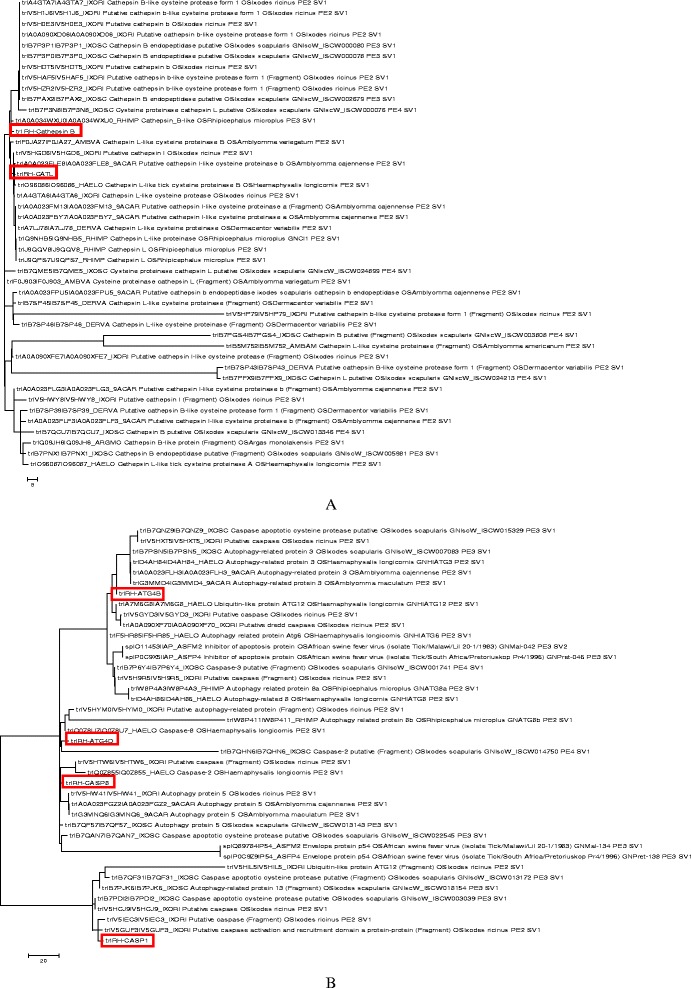
Relationship of *Rhipicephalus haemaphysaloides* cysteine proteases (marked by red box) to other related tick proteins. **a**, the relationship of cathepsins (cathepsin B, cathepsin L and their associated proteins) in ticks. **b**, the relationship of caspases and autophagy proteins in ticks. The circular phylogram is based on the alignment of sequences derived from this study using MEGA by maximum likelihood and similar sequences obtained from the Swiss-Prot database from UniProt

From the transcript library, 6 genes (4 up-regulated genes (CATB, CATL, ATG4B and CASP1) and 2 associated genes (ATG4D and CASP8) were chosen as target genes. They all belong to the cysteine proteases family and the levels of gene expression were detected with RPKM. Although the ticks have great evolutionary distance from other animals, the sequences and phylogram of the target genes showed a close relationship with the cysteine proteases from other species (Fig. 3), suggesting an ancient origin for these genes and a high degree of conservation during evolution. Despite the dispersion of different species, all the sequences in ticks indicate the close relationship among the proteases. The distinction between caspases and autophagy proteases seems to be blurred and indistinct; suggesting that the origin or function of these genes may be closely related in ticks (Fig. 4). All the sequences mentioned above can be found in Additional file 2.

### Dynamic profiling of cysteine proteases in salivary glands of ticks

**Fig. 5:**
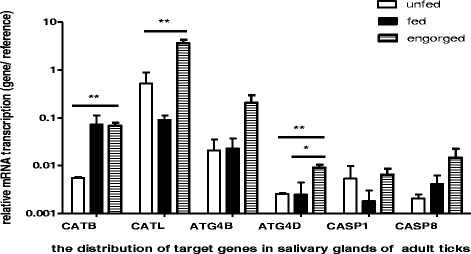
Dynamic cysteine proteases changes in salivary glands of unfed, fed and engorged ticks. Salivary glands were isolated during the different blood feeding statuses and cysteine proteases expression was analyzed by quantitative real-time reverse transcription polymerase chain reaction. Gene expression was calculated using the △Ct method. Elongation factor served as the endogenous control. Data are represented as LS means (*n* = 4 ticks). Significant difference analysis was compared the results of unfed, fed and engorged ticks (*means *P* < 0.05, **means *P* < 0.01, and ***means *P* < 0.001)

Analysis of cysteine proteases mRNA expression in salivary glands is presented in Fig. 5. All the target genes are unregulated after blood feeding and arise their peaks. While the up-regulation genes between unfed and fed are CATB, ATG4B, ATG4D and CASP8, the CATL and CASP1 are down regulated in the fed status. This is different form the report of transcriptome. It seems that the transcriptional levels of 4 up-regulation genes are increased constantly during the blood feeding. CATB, CATL and ATG4D mRNA expression in engorged ticks was significantly higher (*P* < 0.01) than that for unfed ticks.

### Dynamic profiling of cysteine proteases in different developmental stages of ticks

**Fig. 6:**
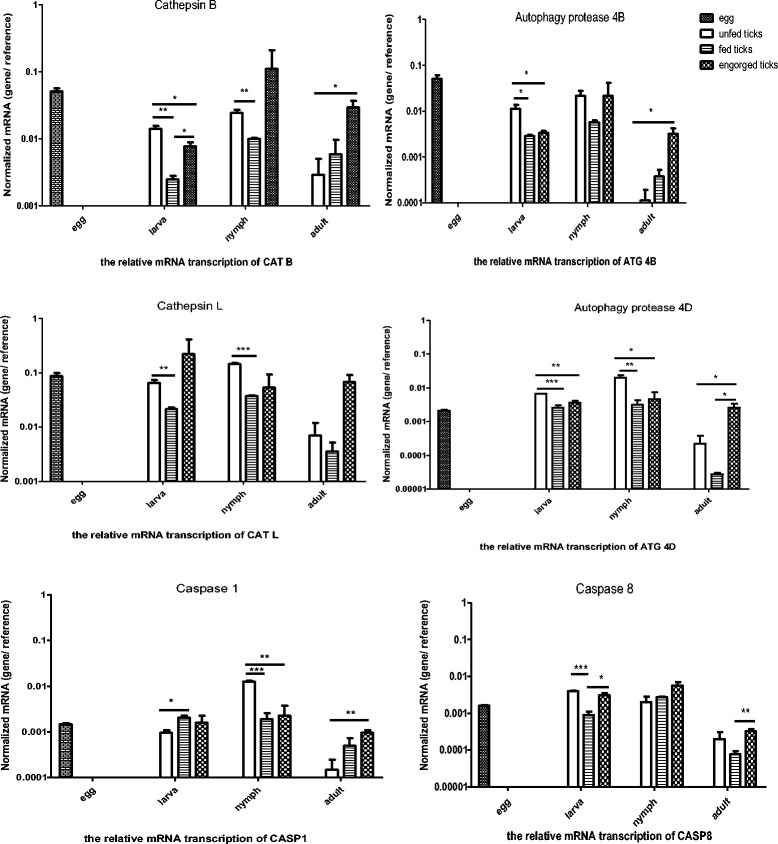
Dynamic cysteine proteases changes in different development of unfed, fed and engorged ticks. Cysteine proteases expression was analyzed by quantitative real-time reverse transcription polymerase chain reaction. Gene expression was calculated using the △Ct method. Elongation factor served as the endogenous control. Data are represented as LS means (*n* = 4 ticks). Significant difference analysis was compared the results of unfed, fed and engorged ticks (*means *P* < 0.05, **means *P* < 0.01, and ***means *P* < 0.001)

Analysis of the mRNA expression of cysteine proteases in egg, larva, nymph and adult is presented in Fig. 6. Almost all of target genes have a down regulation at fed status for the larva and nymph. The same situation occurred for CATL, ATG4D and CASP8 in the adult. CATB appeared to show a declining trend in the larva, and was significantly lower (*P* < 0.01) than that in the unfed ticks. For the nymph, the transcription peak occurred during engorgement, despite its significant reduction (*P* < 0.01) in the fed state. It seems that in the adult the transcription of CATB increases persistently and is significantly higher (*P* < 0.05) than that in the unfed adult. CATL shows a similar tendency to CATB. For the larva and adult, the transcription peak occurred during engorgement. However, the expression in fed larvae and nymphs was significantly lower (*P* < 0.01 and *P* < 0.001, respectively) than that in the unfed stages. ATG4B and ATG4D are regarded as two different isotypes of autophagy-protein associated genes. In the larva and nymph, they appear to show almost the same tendency in unfed, fed and engorged ticks. In the adult, however, ATG4B and ATG4D are at significantly higher levels (*P* < 0.05) than in the unfed tick. This parallels its changes in the salivary glands. The mRNA expression of CASP1 in the larva and adult seems to increase persistently, especially in the adult, where the mRNA expression during engorgement is significantly higher (*P* < 0.01) than that for unfed ticks. However, in fed and engorged nymphs, its transcription is significantly lower than that in unfed nymphs (*P* < 0.001 and *P* < 0.01, respectively). There was low transcription for the mRNA expression of CASP8 in fed larvae and adults, and no obvious regularity was shown in the test.

## Discussions

Most of the proteins displayed in Fig. 3 have been functionally characterized in mammals, while few studies have described the exact function of caspases and autophagy proteases in ticks, although they possess only innate immunity. Almost all the cathepsins in ticks are involved in the process of blood digestion [25]. The peptidases identified from different tick species with a proposed role in hemoglobin digestion are mainly cysteine peptidases, with some serine and metallopeptidases [25, 26]. As in humans, the caspases have the ability to regulate three alternative cell death pathways: apoptosis, pyroptosis, and necroptosis [27]. Moreover, recent work has shown that cathepsin B and cathepsin D regulate the inflammasome-dependent and -independent macrophage responses induced by cytosolic flagellin [28]. The research also revealed that cathepsin B contributes to NAIP5/NLRC4 inflammasome-induced pyroptosis and interleukin-1α (IL-1α) and IL-1β production in response to cytosolic flagellin [28]. Unfortunately, there is little knowledge about the relationships among the cathepsins, caspases and autophagy proteases in ticks. It will be of great value to identify the function of these cysteine proteases because of their important roles, and this may contribute to the development of candidate vaccines or drugs for tick control by RNA interference.

When feeding on their hosts, ticks need to deal with host hemostasis, inflammation and immunity. Although recent progress in transcription research on hard ticks has shown that hundreds of different proteins are expressed in their salivary glands, many of them are known only as salivary proteins with unknown function [8].

Tick feeding is a slow and uninterruptible process, and blood digestion takes place in the gut epithelium, which is different with insect blood-feeders that feed and digest blood in the gut lumen rapidly with neutral pH [29, 30]. In this progress, the salivary glands act as a “pivot” in which anti-host defense molecules and digestion proteases are secreted and released to the host vasculature and to the tick’s midgut with the blood. Pathogens such as *Babesia* [31, 32], *Anaplasma* [33] and *Borrelia* [34, 35] infect tick salivary glands and are injected into the host during their meals [36]. Ticks possess defense mechanisms that allow them to maintain pathogens and commensal microbes at a certain level that does not impair their fitness and further development. Whether any of the proteases also play a role in immune defense is unknown. However, evidence from insects suggests that metalloproteases may be important in cellular immune defense [37]. In mosquitoes, serine proteases are reported to be up-regulated in response to invasion of the hemolymph by malarial parasites, contributing to the normal innate immune response [38].

Cysteine proteases are a component of the multi-enzyme hemoglobinolytic model for hard ticks; its numerous members play different roles in tick life cycles. To our knowledge, CATB and CATL are believed to be involved in the digestion of blood [26]; caspases seem also to participate in this process. In mammals, caspases lead to apoptosis and inflammation and may be associated with autophagy proteases, which are also involved in inflammation and cell death [39]. Unfortunately, their functions in ticks are still unclear. After engorgement, the salivary glands become withered and apoptotic and may even vanish. This process is similar to apoptosis in mammals, but whether caspases and autophagy proteases are involved in this mechanism requires further confirmation.

Cathepsins, another component of the tick multiple enzyme system, are believed to be involved in the digestion of blood. The current knowledge of the molecular characteristics of tick digestive enzymes began to be assembled in the 1980s to 1990s by isolation and partial characterization of acidic aspartic peptidases of cathepsin D from soft and hard ticks [40, 41]. Later, Mendiola et al. reported that aspartic (cathepsin D-like) and cysteine (cathepsin L-like) peptidases are the major hemoglobinolytic enzymes in *R. microplus.* Two cathepsin L-type cysteine peptidases were partially characterized and cloned from the midgut of *Haemaphysalis longicornis* (*H. longicornis*) [42]. Another cysteine peptidase gene homologous to cathepsin L (BmCL1) was shown to be expressed in the gut of partially engorged *R. microplus* females, and recombinant BmCL1 was optimally active against bovine hemoglobin at acidic pH [43, 44]. The research on *Ixodes ricinus* shows more detail about the cathepsins. There is a mechanistic model of the proteolytic pathway of hemoglobin degradation in the digestive vesicles of *I. ricinus* gut cells [26]. Cathepsin D (CATD), supported by cathepsin L (CATL) and legumain (AE), is responsible for the primary events in the cleavage of hemoglobin. Subsequently, cathepsin B (CATB) and cathepsin L (CATL) participate in the secondary digestion, to generate smaller fragments. After the dipeptidase activities of CATB and cathepsin C (CATC), small fragments are degraded into dipeptides. Most CATB and CATL have been identified in the midgut in different tick species: for example, IrCB1 from *I. ricinus* [25, 26, 45], longipain from *H. longicornis* [46], and BmCL1 from *R. microplus* [43, 44, 47, 48]. In *I. ricinus*, IrCL1 was identified in the midgut, salivary glands, ovary and malpighian tubes [26, 49, 50]. Based on our report of the sialotranscriptome, we have identified for the first time that CATB and CATL existed in salivary glands from *R. haemaphysaloides*. These two peptidases may be involved in hemoglobin digestion, and therefore their mRNA expression reaches a peak in engorged ticks and their salivary glands. Given that digestion takes place in the gut epithelium, the midgut of *R. haemaphysaloides* may also contain CATB and CATL. When the blood flows into the gut lumen, it may stimulate and trigger the transcription of peptidases in gut epithelium. Under this circumstance, the mRNA expression in salivary glands may be affected and this may be the reason that there is always low transcription level in the fed state.

Recently, most of the knowledge about caspases has been derived from research on the human or mouse. There is little known about their function in insects, and much less in ticks. However, in-depth analysis on human caspases shows much more detail and offers suggestions for further research in other species. As a member of cysteine proteases family, Caspases have primary specificity for aspartic acid (Asp) residues; they cleave their substrates after tetrapeptide sequences containing Asp in the P1 position. All caspases are synthesized as inactive single-chain zymogens (procaspases) initially, and then processed into their active forms. Additional signals are required to the initiation of caspase activation pathways [27]. Protein interaction domains are component of long prodomains in initiator caspases. For example, CARDs are contained in caspases−1, −2, −4, −5, −9, − 11, and −12, and death effector domains (DEDs) are in caspases-8 and −10 [51]. Caspase−8 as an initiator caspase is activated via their DED-mediated interactions within the death-inducing signaling complex (DISC) in the extrinsic pathway. Ultimately the downstream effector caspases−3, −6, and −7 are activated and responsible for the classical phenotypic changes associated with apoptosis [27]. Caspase-1 is regarded as the prototypical inflammatory caspase [27], and is responsible for the processing of proIL-1β and proIL-18 [52]. Caspase−8 is a mediator of inflammation [27]. Recent studies have revealed distinct roles for caspase−8, which are associated with the extrinsic apoptotic pathway. It is involved in the regulation of inflammation and is also proposed to have an anti-inflammatory role [53]. In addition, caspase−8 is proposed to cleave proIL-1β into its active mature form [54–61], to be incorporated and activated within inflammasome complexes [62, 63], and to activate caspase-1 directly in an inflammasome-independent manner [64, 65].

As mentioned above, it is believed that there is only an innate immune system in ticks. This innate immunity plays really important roles in the tick life cycle, although it may not be as complex and complete as that in mammals. The sialotranscriptome of *R. haemaphysaloides* shows at least 3 or 4 caspases in this tick species, but only caspase−1 and caspase−8 were cloned successfully from the cDNA library. CASP8 reached its peak level after engorgement in our experiment; at this time, ticks are full of host blood, which contains cytokines and perhaps pathogens. As an initiator caspase, CASP8 may trigger and enhance the innate immunity, with CASP1, to defend against harmful effects as described above. The incomplete immune system of the tick may have restricted function when compared with that in mammals. It is still unclear how ticks deal with the cytokines ingested in blood, and whether caspases participate in the management of cytokines and in the mechanism of pathogen defense. Western blot showed that, in female adult *R. haemaphysaloides*, CASP1 may contain ~35 kDa monomers and ~70 kDa dimers (unpublished data), coinciding with that in humans. With reference to studies on human caspases, we will undertake further research on the function of caspases in *R. haemaphysaloides* using RNAi.

Autophagy related (ATG) genes are a complex and mysterious family containing numerous members. There were more than 30 of these genes characterized in yeast originally, and many orthologs have been identified as autophagy regulators in higher eukaryotes [66, 67]. Proteases participate in several stages of autophagy. In the initial steps of macroautophagy, the formation of autophagic vesicles requires the conjugation of phosphatidylethanolamine with ATG8 [68]. Following translation, ATG8 is cleaved by ATG4 (the cysteine family protease) in yeast cells. Subsequently, the resulting ATG8^G116^ is involved in a ubiquitin-like conjugation reaction catalyzed by ATG7 and ATG3 [68]. Similarly, ATG4 participates in processing of three mammalian homologs of ATG8 in mammalian cells, which is crucial for the autophagic pathway [39, 69, 70]. In the sialotranscriptome of *R. haemaphysaloides*, we found only two autophagy-related (ATG) genes; these were classified into ATG4B and ATG4D on the basis of their sequences in BLAST databases. After engorgement, the mRNA expression of ATG4B and ATG4D rises to a peak in adult ticks and their salivary glands, when the salivary glands are approaching apoptosis. It is thought that the ATG-protein may be involved in this process, and that the constitution of autophagosomes in ticks may be much simpler than that in yeast for the limited ATG genes, although its mechanism of action is still unknown.

It is generally suggested that the major function of lysosomal proteases is to maintain cellular homeostasis and differentiation by recycling cellular content [71]. Cathepsin B and Cathepsin L, as the most abundant lysosomal proteases [71], are involved directly in the execution of autophagy [72, 73]. Cathepsin B has also been reported to regulate the activity of caspases [28]. In addition, autophagy-related proteins are acknowledged to be involved in inflammation, infection and cancer [39], meaning that apoptosis and pyroptosis are closely associated with caspases. In view of these findings, it is thought that there must be a closely regulated relationship among the cysteine proteases.

## Conclusions

Analysis of the sialotranscriptome of *R. haemaphysaloides* using two cDNA libraries, from unfed and fed ticks, identified many transcripts coding for different proteins. On the basis of the database of differentially expressed genes, we identified four up-regulated cysteine proteases and two associated genes for further study. Using Q-PCR, the up-regulated genes were found to be CATB, ATG4B, ATG4D and CASP8, which is slightly different from results of the sialotranscriptome, although mRNA expression of all six target genes reached a peak after engorgement. Moreover, the phylogenetic trees showed that the *R. haemaphysaloides* cysteine protease sequences are dispersed into different clades, which contain sequences from other species, suggesting an ancient origin for these genes. The phylogram of different tick species demonstrates the close relation between caspases and autophagy-related proteins. Furthermore, we observed dynamic changes of mRNA expression in different developmental stages of *R. haemaphysaloides*, suggesting their multiple functions during blood feeding.

Taken together, these results improve our knowledge of the salivary components and distribution of cysteine proteases of *R. haemaphysaloides* that will make a contribution to further study, and may help in the research to identify candidate antigens for anti-tick vaccines and to discover drugs to treat inflammation and cancer.

## Ethics statement

The protocols were approved by the Institutional Animal Care and Use Committee of the Shanghai Veterinary Research Institute, and followed the misconduct policy of BMC Genomics, and authorized by the Animal Ethical Committee of Shanghai Veterinary Research Institute.

## Additional files

Additional file 1:
**Differentially expressed genes database and GO terms database involved in this paper.** (ZIP 4091 kb) Additional file 2:
**Details of all the proteins involved in the phylogenetic analysis of this paper.** The text contained all the cysteine proteases form different species involved in this paper, including the proteases form *R. haemaphysaloides*(RH-) which were confirmed by the experiments. (TXT 65 kb)
